# Assessment of the WAP-Myc mouse mammary tumor model for spontaneous metastasis

**DOI:** 10.1038/s41598-020-75411-z

**Published:** 2020-10-30

**Authors:** Begüm Utz, Rita Turpin, Johanna Lampe, Jeroen Pouwels, Juha Klefström

**Affiliations:** Cancer Cell Circuitry Laboratory, Translational Cancer Medicine Research Program, Research Programs Unit, Faculty of Medicine, University of Helsinki, Helsinki, Finland

**Keywords:** Breast cancer, Cancer models, Cancer microenvironment

## Abstract

Breast cancer is the most common form of cancer in women. Despite significant therapeutic advances in recent years, breast cancer also still causes the greatest number of cancer-related deaths in women, the vast majority of which (> 90%) are caused by metastases. However, very few mouse mammary cancer models exist that faithfully recapitulate the multistep metastatic process in human patients. Here we assessed the suitability of a syngrafting protocol for a Myc-driven mammary tumor model (WAP-Myc) to study autochthonous metastasis. A moderate but robust spontaneous lung metastasis rate of around 25% was attained. In addition, increased T cell infiltration was observed in metastatic tumors compared to donor and syngrafted primary tumors. Thus, the WAP-Myc syngrafting protocol is a suitable tool to study the mechanisms of metastasis in MYC-driven breast cancer.

## Introduction

Breast cancer (BCa) is a global health problem, as it is the most common cancer among women^1^. The vast majority of BCa deaths (> 90%) are caused by metastases^2^, which develop in a multi-step process that starts with the local invasion and migration of tumor cells, which then intravasate into lymphatic or blood vessels. These circulating cancer cells can reach distant organs and form metastatic tumors at secondary sites^3^, most commonly bone, lung, liver and brain in case of breast cancer^4^. Understanding the biological mechanisms that drive distant colonization and metastatic tumor formation could enable the development of therapeutic approaches and improve patient outcome.

Mouse models are fundamental for preclinical studies of tumor biology and cancer therapy. Several strategies have been devised to develop mouse models of metastatic breast cancer; however, these models have shortcomings in recapitulating the multistep metastatic process in human patients^5–7^. The commonly-used experimental metastasis model involves injection of tumor cells into the tail vein, which recapitulates the distant seeding and colonization of cancer cells but omits the local invasion and intravasation steps^8^. Therefore, it does not reflect all stages of the metastatic process. Another common method to model metastasis is the injection of highly metastatic mouse mammary tumor cells into the mammary fat pad of a syngeneic recipient mouse. 4T1 cells, derived from a spontaneous mammary tumor in the BALB/c genetic background, or Met-1 cells, derived from a MMTV-PyMT mammary tumor in FVB/N background, are frequently used in this context as they can metastasize to multiple distant organs in relatively short time^9,10^. The main disadvantage of using these cell lines is that they do not represent the heterogeneity of BCa, making them less than ideal models to study therapeutic responses^11^. Genetically-engineered mouse models (GEMMs) of breast cancer overcome many of these issues and they closely recapitulate the human cancer histopathology and progression^12^. However, GEMMs develop metastatic tumors with varying frequencies, and in an asynchronous manner, making their use challenging^13^. Furthermore, these mice usually succumb to the rapidly growing primary tumor before prominent metastatic disease occurs.

Syngraft models, where tumor cells or fragments isolated from GEMMs are orthotopically transplanted to syngeneic recipient mice, present a promising approach to develop a metastatic mouse model, especially when the primary tumor gets surgically resected to allow time for metastatic tumors to develop^7^. This approach has been successfully used to model invasive lobular breast cancer with the conditional K14cre;Cdh1F/F;Trp53F/F mouse model, which lacks E-cadherin and p53 expression in the mammary epithelium, and develops de novo mammary tumors from 4 months of age^14^. Tumor fragments isolated from these mice were orthotopically transplanted to syngeneic recipient mice, and when the primary tumors reached a predetermined size, they were surgically removed, mimicking mammary tumor resection as is routinely performed in the clinic. The removal of the primary tumor allowed time for metastatic tumors to spontaneously develop in many organs within 2–6 months following surgery. This metastatic mouse model is superior to others mentioned above as it closely recapitulates the course of the metastatic disease in the clinic. However, it should be noted that the development of metastatic tumors takes a long time (2–6 months), making routine applications time-consuming and costly. Nevertheless, applying this approach in other GEMMs of BCa could yield metastatic models of breast cancers driven by diverse genetic changes, increasing our understanding of metastasis in different contexts.

C-MYC is frequently amplified and/or overexpressed in human breast cancers and is associated with poor outcome and metastasis^15^. Through regulation of critical cellular processes, including ribosome biogenesis, translation, cell cycle and metabolism, MYC overexpression confers a growth advantage to tumor cells^16–18^. As MYC-overexpressing tumor cells depend on MYC for survival and proliferation, MYC has been a compelling target for therapeutic intervention^19,20^. Although targeting MYC directly has been difficult as it is a transcription factor without enzymatic activity, several attempts have been made^21–23^. As an alternative, new strategies have been developed to target MYC-driven oncogenic processes^21,24^. For example, using a transgenic model for MYC-driven mammary cancer (the WAP-Myc mouse model), we recently identified MYC-driven anti-apoptotic pathways as a vulnerability in MYC-high breast cancer^25^. In the WAP-Myc mouse model, MYC is overexpressed in the mammary gland under the whey acidic protein (Wap) promoter resulting in the development of breast adenocarcinomas with a latency of 2–3 months^26^. However, while WAP-Myc mice are an excellent model to investigate therapeutic approaches against MYC, their use has been limited to studies of primary tumors as WAP-Myc tumors rarely metastasize^27^. In this study, we investigated whether the WAP-Myc model can be developed into a model for spontaneous metastasis of MYC-driven breast cancer by applying a recently developed protocol for WAP-Myc syngrafting and resection of the primary tumor generated from syngrafted cells^25^.

## Material and methods

### Mice

WAP-Myc mice (FVB.Cg-Tg(Wap-Myc)212Bri/J) and Luciferase-GFP reporter mice FVB-Tg(CAG-luc,-GFP)L2G85Chco/J were obtained from the Jackson Laboratory (Bar Harbor, Maine, United States)^27,28^. Genotyping was conducted by PCR analysis following the protocols of the Jackson Laboratory. To generate the FVB/N-tg(Wap-Myc; CAG-luc,-GFP) transgenic mice, 8–10 weeks old WAP-Myc males and Luc;GFP females were crossed and the heterozygous offspring from different crossings were bred to obtain homozygous Wap-Myc;Luc;GFP transgenic mice. 4 week-old, wild-type FVB mice were purchased from Janvier Labs (Le Genest-Saint-Isle, France). All experimental procedures involving animals were approved by the National Animal Ethics Committee of Finland (License number: ESAVI/3678/04.10.07/2016), and mouse colonies were maintained in accordance with the protocols of the Experimental Animal Committee of the University of Helsinki.

### Isolation of donor tumor cells

To induce MYC expression under the Wap promoter, WAP-Myc or WAP-Myc;Luc;GFP females underwent two pregnancies, after which mice were monitored weekly for mammary tumor formation. When tumors were measured to be approximately 10 mm × 10 mm in dimensions, tumors were harvested into an ice-cold solution of PBS. In a laminar hood, PBS was removed and tumors were mechanically minced using sterile scalpels. Tumor pieces were then transferred into DMEM/F12 medium containing 5% FCS, 200 mM L-glutamine, 5 μg/ml insulin, 50 μg/ml gentamycin and 0.2% collagenase A (w/v) and incubated at 37 °C for 2 h with moderate shaking (120 rpm). Samples were washed four times with DMEM/F12 medium without collagenase A and digested further with trypsin for 10 min at 37 °C. Following a wash with complete DMEM/F12 medium, cells were counted and either stored frozen in liquid nitrogen, cultured overnight or transduced with a lentiviral vector to express luciferase. Lentiviral particles were prepared using the pLenti CMV Puro LUC (w168-1) plasmid which was a gift from Eric Campeau and Paul Kaufman (Addgene plasmid #17477; https://n2t.net/addgene:17477; ^29^).

### Orthotopic transplantation of tumor cells

4-week-old FVB female recipient mice were injected with painkillers (carprofen and buprenorphine) and anesthetized using inhaled 2.5% isoflurane. After disinfection, a midline incision was made and the 4th mammary glands were exposed. 25,000–100,000 WAP-Myc tumor cells, resuspended in 10 μl PBS containing 1% FBS, were injected bilaterally into the 4th mammary glands and the skin was sealed with wound clips. The mice were monitored after surgery to ensure well-being.

### Surgical resection of tumors

Primary tumor size was measured three times a week using digital calipers. Tumor volumes were calculated using the formula V = d2 × D/2, where d represents the shortest diameter and D represents the longest. When one of the bilateral tumors reached 750 mm^3^ in volume, both tumors were surgically resected under isoflurane anesthesia under sterile conditions. The incision was closed using wound clips. Mice were given painkillers (carprofen and buprenorphine) to alleviate pain and monitored after surgery to ensure well-being. After surgery, the mice were followed for secondary tumor formation or signs of metastatic disease. Mice were sacrificed either when the diameter of the secondary tumors reached 1.5 cm or when they showed signs of metastatic disease, as indicated by difficulty breathing or weight loss.

### Tumor immunoprofiling

0.1 g of the resected tumors was digested in DMEM/F12 medium containing 2 mg/ml collagenase A for 1.5 h at 37 °C on a shaker. After washing, samples were further digested in TrypLE express (12604013, ThermoFisher) for 10 min at 37 °C. Samples were filtered through a 70 μm cell strainer, split into three separate tubes and stained for 15 min in room temperature with the antibody panels listed in Table 1. Samples stained with antibody panel 2 were fixed and permeabilized using Fixation/Permeabilization Solution kit (BD) as some of the target proteins are intracellular.

**Table 1:** Antibody panels used in tumor immunoprofiling.

Panel 1	CD45–APC Cy7 (103116, Biolegend), CD3–APC (100236, Biolegend), CD4–V500 (560782, BD), CD8–FITC (11-0083-85, Invitrogen), NK1.1–V450 (clone PK136560524, BD), CD107–PE (121611, Biolegend), PD1–PerCP Cy.5 (clone 29F.1A12, 135208, Biolegend)
Panel 2	CD45–APC Cy7 (103116, Biolegend), CD3–APC (100236, Biolegend), CD4–V500 (560782, BD), CD8–FITC (11-0083-85, Invitrogen), NK1.1–PE (557391, BD), Intracellular markers: interferon gamma–BV421 (563376, BD), Granzyme B–PE Cy7 (25889882, Biolegend)
Panel 3	CD45–PE (clone 30-F11, 553081, BD), CD11b–BV510 (clone M1/70, 562950, BD), Ly6G–PerCP Cy5.5 (560602, BD), Ly6C–Alexa Fluor 700 (561237, BD), PDL1–BV421 (clone MIH5, 564716, BD), CD86–APC (clone GL1, 558703, BD), CD80–PE Cy7 (clone 16–10A1, 104734, Biolegend)

The samples were analyzed using FACSVerse (BD) and analyzed using Flowjo version 10.4. To analyze panels 1 and 2, cells were first gated for singlets, then CD45 + leukocytes were selected. Leukocytes were assessed by the expression of CD3 and NK1.1 to identify T cells (CD3 + NK1.1−), NK cells (NK1.1 + , CD3−) and NKT cells (CD3 + NK1.1 +). T cells (CD3 +) were further gated to classify T helper cells (CD4 +) or cytotoxic T cells (CD8 +). Expression of CD107, interferon gamma, PD-1 (programmed cell death-1) and granzyme B was measured as a marker for T cell activity. To analyze panel 3, cells were first gated for singlets and CD45 + positivity. CD11b + and Ly6G cells were classified as neutrophils, while CD11b + and Ly6C + cells classified monocytes.

### Immunohistochemistry

Tissue material was collected from the indicated tissues, fixed with 4% paraformaldehyde and embedded in paraffin. 5 µm sections were cut, deparaffinized and treated with 1 × Antigen retrieval citrate buffer solution (Dako) for 20 min in a UPO 1330G microwave set to 40% power. Immunohistochemistry was carried out as described^25^. The following primary antibodies were used: c-MYC (clone Y69, ab32072, Abcam), Ki67 (ab15580, Abcam), ColIV (ab6586, Abcam), Cytokeratin 14 (#905301, Biolegend) and CD3 (clone D7A6E, Cell Signaling Technology). Samples were imaged using a 20X objective on a Leica DM LB microscope with Studio-Lite 1.0 software (Biomedicum Imaging Unit, University of Helsinki). Whole slide scanning was done using 3DHistech Panoramic 250 FLASH II digital slide scanner (Genome Biology Unit, University of Helsinki).

### Data analysis

Data analyses and statistics were done using GraphPad Prism 8 software. Details of statistical tests used are listed in the figure legends. In short, paired t-test was used to compare the primary and metastatic tumor samples from the same mouse. Student’s t-test was used for other calculations. A p-value under 0.05 was considered statistically significant and denoted with an asterisk (*). Graphs were prepared using GraphPad Prism or www.draw.io website. Data are represented as mean ± standard deviation (s.d.). Figures were prepared in Adobe Illustrator CC 2019.

## Results

### Development of the transplantation and tumor resection protocols for the WAP-Myc metastatic mouse model

**Figure 1:**
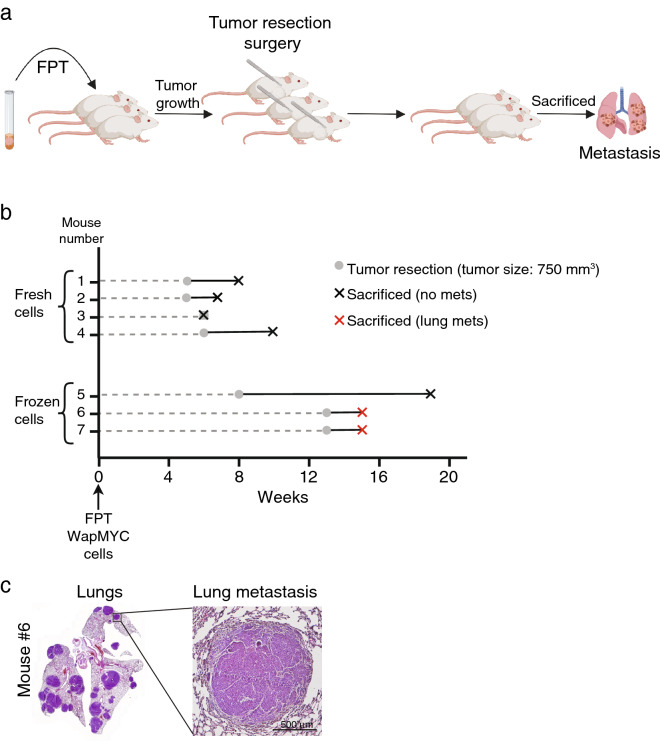
Generation of the MYC-driven, metastatic breast cancer mouse model with spontaneous lung metastasis. (**a**) Schematic representation of the experimental approach to generate the WAP-Myc metastatic mouse model. WAP-Myc tumor cells were isolated from donor mice and then transplanted bilaterally to the 4th mammary fat pads of 4-week old recipient mice (*FPT* fat pad transplantation). Primary tumors were surgically resected at 750 mm^3^ to allow more time for metastases to form. Mice were sacrificed upon signs of metastatic disease or local relapse of the resected tumors. (**b**) Timeline of the optimization experiment for each individual mouse. At week 0 mice were transplanted with either fresh or previously-frozen WAP-Myc tumor cells. Gray circles indicate the time point when the primary tumor was surgically removed. Crosses indicate the time each mouse was sacrificed; a black cross indicates lack of metastasis at the time of sacrifice, while a red cross indicates the presence of lung metastases. (**c**) Left: Whole-slide scan of the H&E-stained lungs from mouse #6 from the experiment in (**b**). Right: a close-up of one of the metastatic nodules. Scale bar = 500 μm.

To develop a metastatic mouse model of MYC-driven breast cancer, we used transgenic WAP-Myc mice, which develop breast adenocarcinomas following two pregnancies. As these mammary tumors grow relatively fast, metastatic tumors are rare when the mice are sacrificed due to primary tumor burden^27^. In an attempt to increase the frequency of metastasis, WAP-Myc tumor cells were orthotopically syngrafted to recipient mice and, to give more time for metastatic tumors to develop, primary tumors were surgically resected after reaching a volume of 750 mm^3^ (Fig. 1a), similar to the approach taken by Doornebal et al. for the conditional K14cre;Cdh1F/F;Trp53F/F mouse model^14^. Throughout the remainder of the paper, for syngrafting experiments the term primary tumor is used to indicate the primary tumor generated from syngrafted cells and the term donor tumor indicates the tumor the syngrafted cells were derived from.

To establish the protocol we performed a preliminary experiment where either 100,000 fresh (never frozen) or 200,000 frozen-and-thawed donor tumor cells were syngrafted into the fat pads of both 4th mammary glands in recipient mice (eight mice in total). Donor tumor cells were transduced with CMV-Puro-Luc vector prior to implantation to monitor primary and metastatic tumor growth but, while bioluminescence signals could be detected for primary tumors, we could not detect them for metastatic tumors (data not shown). Primary tumors were resected when one of them reached 750 mm^3^, which took 5–6 weeks for tumors grown from fresh cells and 8–13 weeks for tumors from frozen cells (Fig. 1b). One mouse had to be sacrificed before any signs of primary tumor growth and was eliminated from analysis. After tumor resection, mice were sacrificed upon growth of secondary mammary tumors with a diameter of 15 mm or signs of discomfort due to metastasis, after which the mice were surveyed for metastasis in the lungs, liver, brain, lymph nodes and spleen. Spontaneous lung metastases were detected in two out of seven mice, both transplanted with frozen-and-thawed cells (Fig. 1b,c). None of the mice developed detectable metastases in other organs.

Despite the low number of mice, this preliminary experiment suggests that in this setup frozen-and-thawed donor tumor cells are more likely to form metastases than fresh donor tumor cells (Fig. 1b). The delay in primary tumor growth with frozen-and-thawed cells (Fig. 1b) could provide a longer window for metastatic cells to disseminate and for metastases to form.

### Increased survival time after tumor resection, but not time to primary tumor resection, correlates with metastatic occurrence

**Figure 2:**
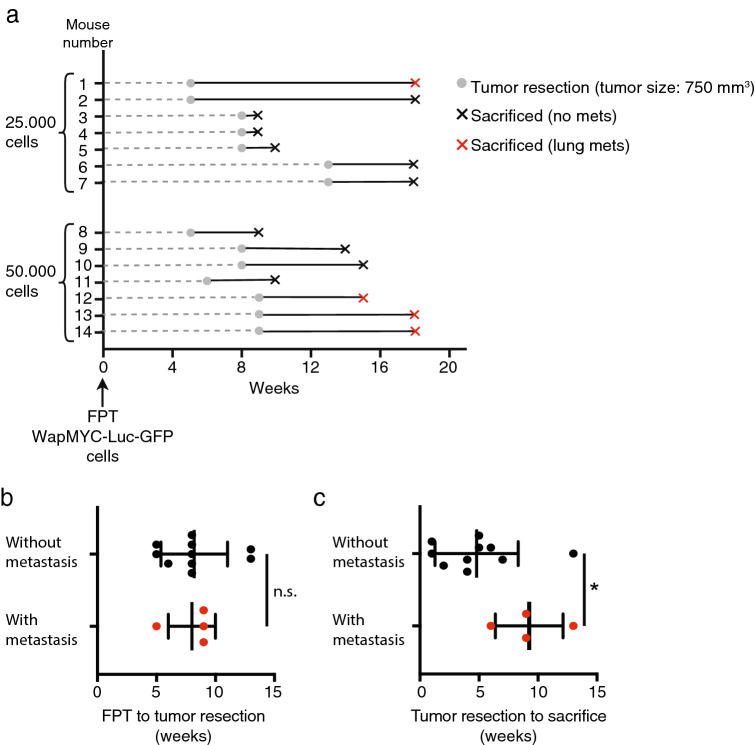
Metastatic tumors occur in 28% of mice with syngrafted WAP-Myc tumors after primary tumor surgery. (**a**) The timeline of the experiment for each individual mouse. Mice were transplanted (*FPT* Fat pad transplantation) with 25,000 or 50,000 WAP-Myc tumor cells at week 0. Gray circles indicate the time point when the primary tumor was surgically removed. Crosses indicate the time each mouse was sacrificed; a black cross indicates lack of metastasis at the time of sacrifice, while a red cross indicates the presence of lung metastases. (**b**,**c**) The time from fat pad transplantation to tumor resection (**b**) or from tumor resection to sacrifice (**c**) in mice that developed metastasis vs. mice that did not develop metastasis. (n = 14 mice, 10 mice that did not develop metastases and 4 that did; n.s. not significant, *p = 0.045; Student’s t test).

As luciferase could not be detected in metastatic tumors when transduced WAP-Myc cells were transplanted, we developed a new transgenic luciferase expressing WAP-Myc mouse line (FVB/N-tg(Wap-Myc; CAG-luc,-GFP)) by crossing WAP-Myc mice with FVB/N-tg(CAG-luc,-GFP) mice^28^. Donor tumors developed with similar kinetics in these mice as in WAP-Myc mice (data not shown). Our preliminary experiment (Fig. 1b) suggested that freezing and thawing cells prior to transplantation correlates with increased metastatic tumor development. Therefore, frozen-and-thawed FVB/N-tg(Wap-Myc; CAG-luc,-GFP) donor tumor cells were syngrafted. In addition, as increased time until tumor resection correlates with increased occurrence of metastasis^30^, we decreased the number of syngrafted cells to 25,000 and 50,000 (eight mice each). Two mice (one in each group) had to be sacrificed during the course of the experiment for reasons unrelated to WAP-Myc tumorigenesis and were eliminated from analysis. Primary tumors were surgically removed when one of the tumors reached 750 mm^3^, which happened at 5–13 and 5–9 weeks after syngrafting in mice with 25,000 and 50,000 syngrafted cells, respectively (Fig. 2a). At the endpoint, 4 out of 14 mice had developed lung metastasis (28%; Fig. 2a). The time frame between fat pad transplantation to primary tumor resection did not affect the chance of metastasis (Fig. 2b). However, prolonged time between tumor resection and sacrifice positively correlated with the likelihood of metastatic growth (Fig. 2c). We could not detect any metastatic tumors through in vivo bioluminescence imaging, despite the facts that lung metastases were fairly large and the primary tumor was detectable (data not shown).

### Metastatic tumors maintain the histopathological features of the donor tumor

**Figure 3:**
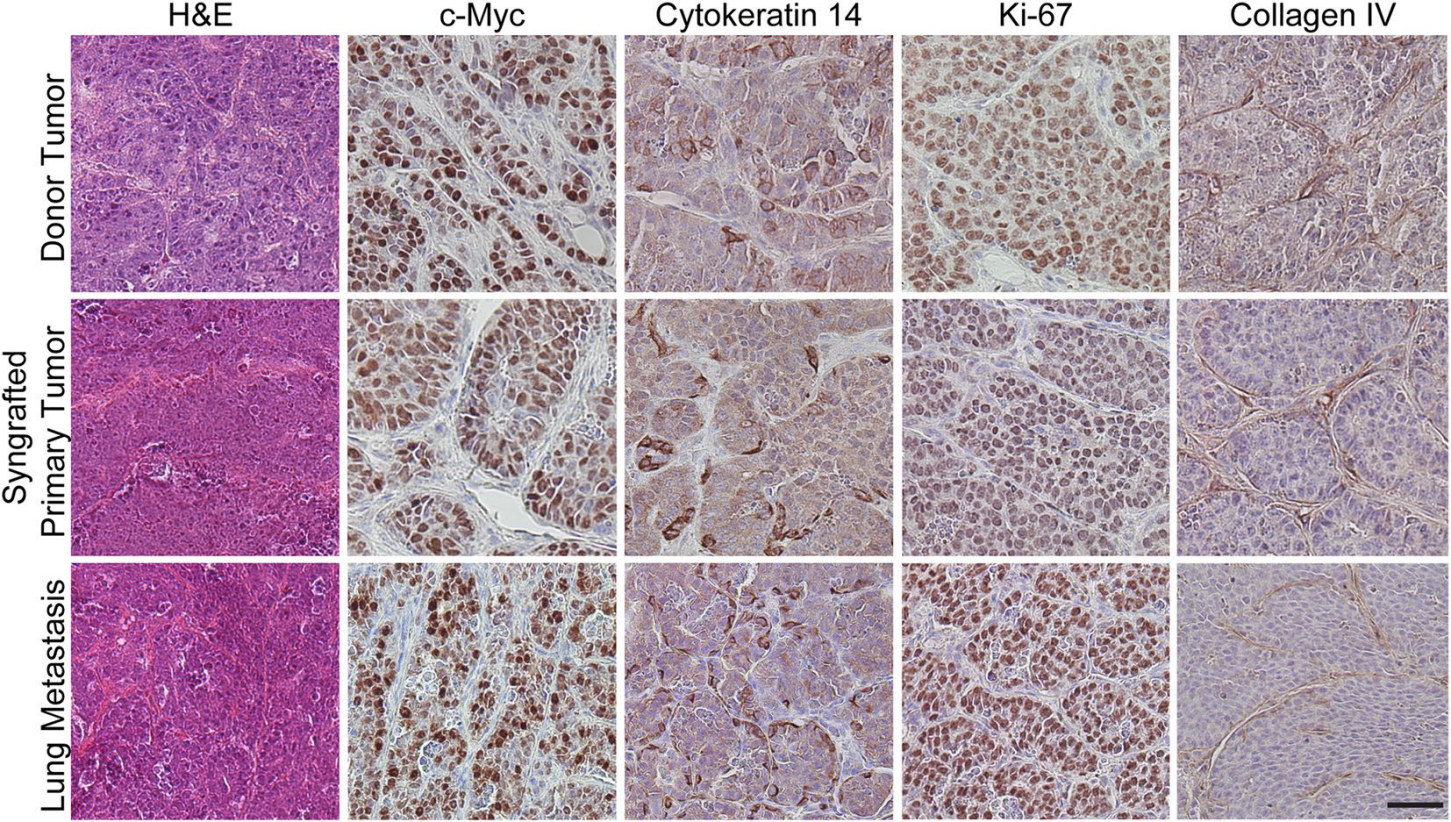
Histopathological features are similar in WAP-Myc mammary donor tumors, syngrafted primary tumors and metastatic lung tumors. Representative immunohistochemistry images from FVB/N-tg(Wap-Myc; CAG-luc,-GFP) mammary donor tumors, syngrafted primary tumors and metastatic lung tumors, stained as indicated. Scale bar = 50 μm.

We next compared the histopathological characteristics of the FVB/N-tg(Wap-Myc; CAG-luc,-GFP) mammary donor tumors to the syngrafted primary tumors and metastatic lung tumors (Fig. 3). Syngrafted primary tumors and metastatic lung tumors retained the strong c-myc expression of donor tumors. Cytokeratin 14, a marker for myoepithelial cells, and collagen IV, a marker for basal lamina, were expressed to similar levels and with similar localization in all tumors. In addition, all tumors were highly proliferative, as evidenced by the expression of Ki67 antigen. Thus, metastatic WAP-Myc tumors recapitulate the morphological characteristics of syngrafted primary tumors, which closely mimic the histopathology of donor tumors.

### Metastatic WAP-Myc tumors contain increased numbers of tumor-infiltrating T cells compared to primary tumors

We previously showed that apoptosis in WAP-Myc tumors, induced with venetoclax and metformin treatment, triggered an anti-tumor immune response, and we identified a role for T cells in this response^25^. To gain insight into the immune contexture of WAP-Myc metastatic tumors, we immunoprofiled 6 matching primary and metastatic lung tumors from two independent experiments using flow cytometry analyses. Four tumor pairs were from the experiment with FVB/N-tg(Wap-Myc; CAG-luc,-GFP) mice (Figs. 2 and 3). The other two tumor pairs were from an experiment with the same experimental setup, except that donor tumor cells from WAP-Myc mice (without GFP;Luc) were syngrafted. In the latter experiment, 25% (2 out of 8; time line data not shown) of mice syngrafted with WAP-Myc donor tumor cells formed metastatic tumors, similar as in the experiment with WAP-Myc;GFP;Luc mice (28%; Fig. 2a).

**Figure 4:**
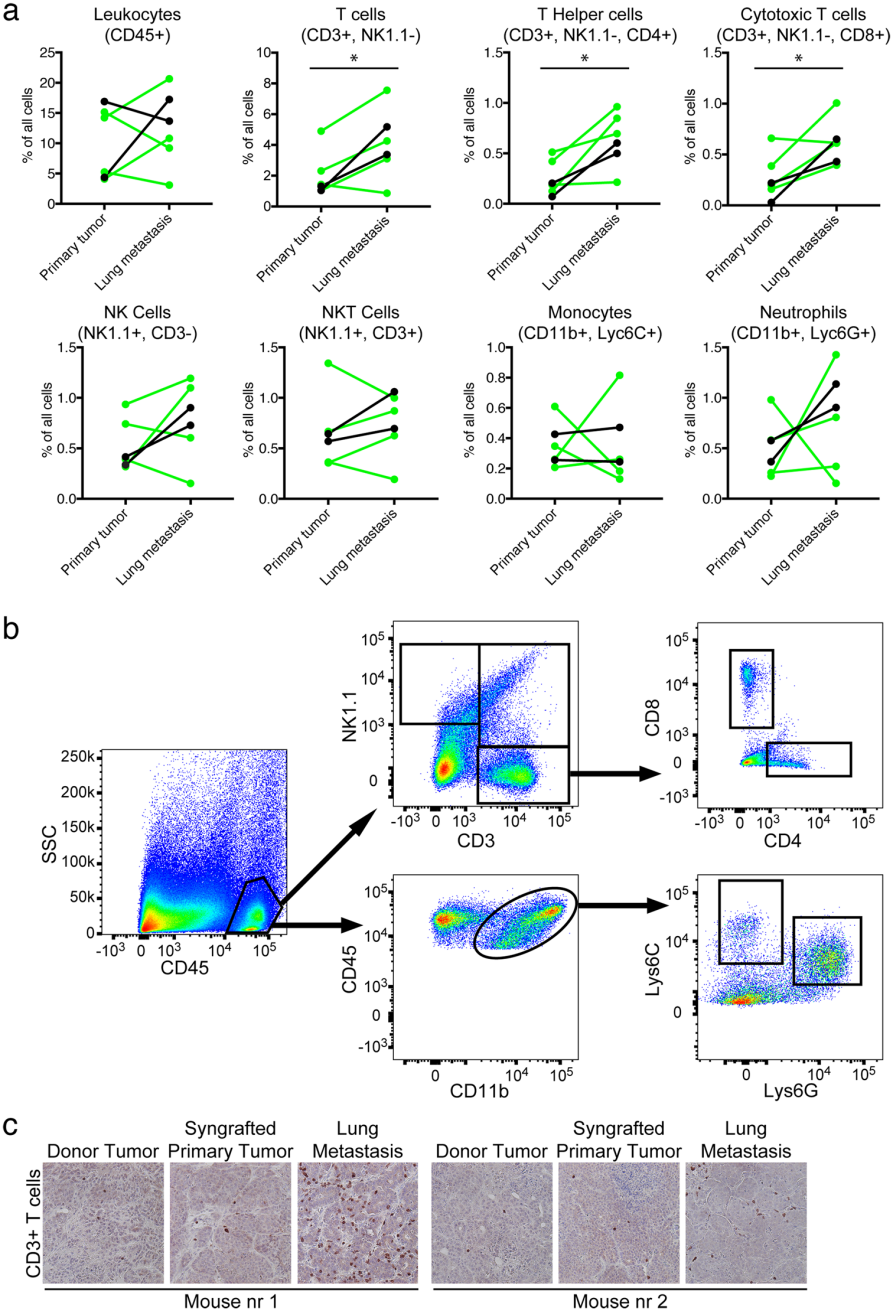
Metastatic lung tumors have higher T-cell infiltration compared to primary tumors. (**a**) Flow cytometry analyses of the number of leukocytes (CD45 +), total T cells (CD3 +), T helper cells (CD3 + CD4 +), cytotoxic T cells (CD3 + CD8 +), NK cells (CD3− NK1.1 +), NKT cells (CD3 + NK1.1 +), monocytes (CD11b + Ly6C +) and neutrophils (CD11b + Ly6G +) in 6 matching primary and metastatic lung tumors. Four tumor pairs were from mice transplanted with WAP-Myc;GFP;Luc cells (as in Figs. 2, 3) and two tumor pairs were from mice transplanted with WAP-Myc cells (indicated in green and black, respectively). A paired t-test was used to compare primary and metastatic lung tumors (* p < 0.05; CD3 + cells, p = 0,0215; CD4 + cells p = 0.0154; CD8 + cells p = 0.0235). (**b**) Flow cytometry gating strategies for the immune cell populations quantified in (**a**). Gating examples are from the primary tumor from the same mouse. (**c**) Representative immunohistochemistry images of donor tumor slices from two mice and matched syngrafted primary tumor and lung metastasis stained for T cell marker CD3. Scale bar = 50 μm.

**Figure 5:**
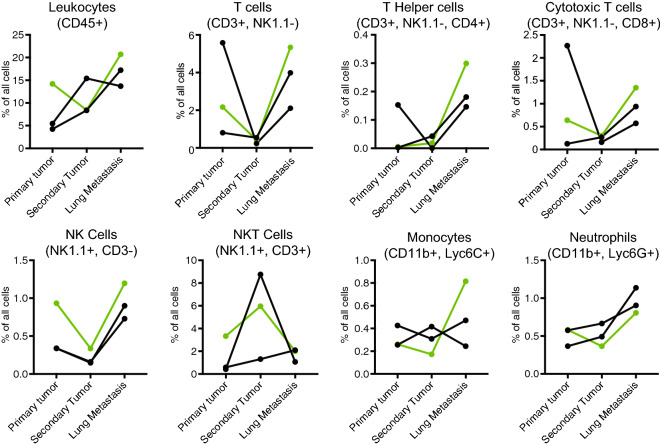
Metastatic lung tumors have higher T-cell infiltration compared to secondary tumors. (**a**) Flow cytometry analyses as in Fig. 4a in 3 matching primary, secondary and metastatic lung tumors. One tumor pair was from mice transplanted with WAP-Myc;GFP;Luc cells and two tumor pairs were from mice transplanted with WAP-Myc cells, indicated in green and black, respectively.

**Figure 6:**
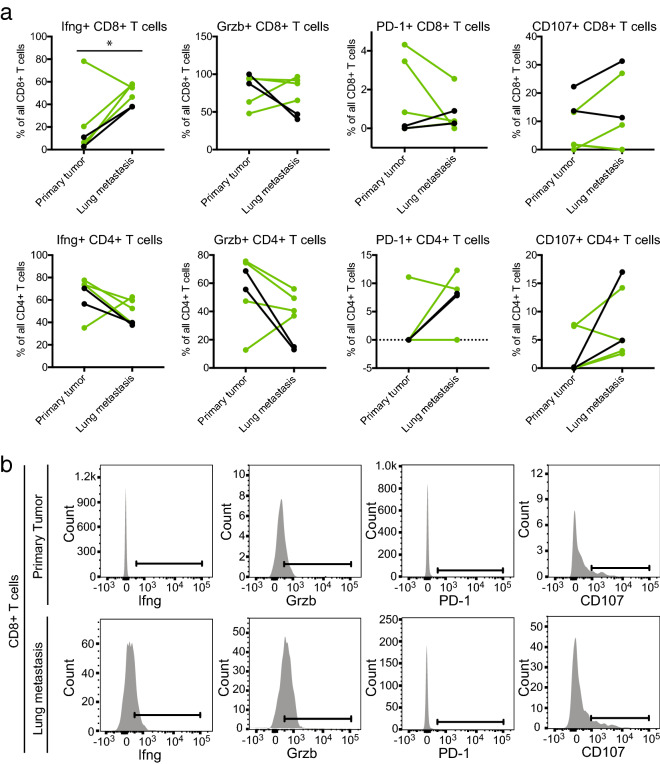
Higher interferon gamma expression in cytotoxic T cells (CD8 +) in lung metastases compared to matching primary tumors. (**a**) Flow cytometry analyses of PD-1, CD107a, Granzyme B and interferon gamma expression in T helper cells (CD3 + CD4 +) and cytotoxic T cells (CD3 + CD8 +) in the same 6 matching primary and metastatic lung tumors as analysed in Fig. 4a. Four tumor pairs were from mice transplanted with WAP-Myc;GFP;Luc cells (as in Figs. 2, 3) and two tumor pairs were from mice transplanted with WAP-Myc cells (indicated in green and black, respectively). A paired t-test was used to compare primary and metastatic lung tumors (* p = 0,0494). (**b**) Flow cytometry gating strategies for the markers quantified in (**a**) as exemplified using cytotoxic T cells (CD8 +) from metastases and primary tumor samples from the same mouse.

Immunoprofiling data from these tumor pairs are shown in Figs. 4, 5, 6. We observed significantly higher T cell infiltration (both cytotoxic T (CD8 +) cells and T helper (CD4 +) cells) in metastatic tumors compared to primary tumors from the same mouse, whereas infiltration of other immune cell types did not show significant differences (Fig. 4a,b). Immunohistochemical staining for CD3, a marker of all T cells, confirmed the increased T cell infiltration in metastatic tumors compared to donor and syngrafted primary tumors (Fig. 4c). Immunoprofiling of secondary tumors, which had formed in 3 out of 6 mice with metastasis at the end of the experiment, revealed lower T cell infiltration in secondary tumors compared to metastases (Fig. 5), suggesting that the increased T cell infiltration in metastatic tumors is specific and not due to systemic changes, for example due to mouse development. Staining for T cell activity markers showed that cytotoxic T cells (CD8 +) infiltrated in lung metastases expressed more interferon gamma expression compared to cytotoxic T cells in the matching primary tumors (Fig. 6). No other significant changes in T cell activity markers were observed between metastases and primary tumors (Fig. 6).

## Discussion

MYC is a potent oncogene that drives tumor initiation and maintenance. As MYC is frequently amplified or overexpressed in high-grade breast tumors^25,31^, substantial efforts have been put forth to target MYC, either directly^21–23^ or by exploiting MYC-induced cancer vulnerabilities^21,24,25^. Therefore, a need exists for preclinical models to study the therapeutic response to MYC targeting in vivo. While recent papers describe spontaneous progression to metastasis in MYC-driven KRAS^G12D^ models of lung^32^ and pancreatic cancer^33^, to the best of our knowledge, there are no MYC-driven models of breast cancer to represent advanced, metastatic tumors. A few mouse models of breast cancer are driven by MYC, such as WAP-MYC and MMTV-MYC, but they are poorly metastatic^27,34,35^. In this study, we aimed to generate a clinically-relevant, MYC-driven metastatic breast cancer mouse model by syngrafting WAP-Myc tumor cells to syngeneic recipient mice. To extend the survival of the syngrafted mice and thus increase the window of opportunity for tumor cells to establish metastases, the primary tumor was removed via surgical resection when it reached 750 mm^3^. This experimental approach has been successfully applied to generate metastatic mouse models using other transgenic mouse lines^7,14^. We found that survival time from tumor resection to disease progression correlated with likelihood of metastatic spread, which occurred in around 25% of the mice. It is well-established that the microenvironment plays a critical role in metastatic processes^36^, and it should be pointed out that our syngrafting experiments were performed with heterogenous tumor material including tumor cells, mesenchymal and residual fat cells, and immune cells. For example, a study by Ricke and coworkers^37^ showed that while small non-invasive tumors developed when immortalized prostatic epithelial cells were transplanted alone, tumors with metastatic ability were generated only after transplantation of mice with mesenchymal cells and immortalized prostatic epithelial cells. Therefore, it is highly likely that at least part of the metastatic capacity in syngrafted tumors involves the stromal component of the tumor syngrafts. Given that injection of tumor cells into tail veins of mice commonly leads to lung metastases, we cannot fully exclude the possibility that some metastases were contributed by direct leakage of transplanted cells into the bloodstream through microvessels. This is unlikely, however, given that transplantation of 100,000 fresh cells (Fig. 1) gave rise to fewer metastases than transplantation of 25,000 or 50,000 fresh cells (Fig. 2) and injecting breast cancer cells in the mouse mammary fat-pad is a relatively simple procedure unlikely to result in significant tumor cell leakage^38,39^.

The overall metastasis rate of 25% is unfavorable to reliably assess the effects of different drugs on metastasis, as very large numbers animals would need to be used in one experiment. For example, in order to reliably obtain eight mice with metastasis, more than 32 mice have to be transplanted per treatment group, thus requiring large quantities of mice and drugs. However, the WAP-Myc metastatic breast cancer model described here can be a valuable tool to study the mechanism of metastasis in aggressive MYC-high breast tumors. The advantage of the WAP-Myc model is that it maintains all the crucial biological steps of the metastatic process and closely mimics the clinical progression of metastatic breast cancer in patients. To increase the metastatic frequency, tumor cells from these spontaneous metastatic tumors can be passaged in vivo through serial transplantations to select highly metastatic variants^40,41^. However, these variants may lack the heterogeneity of the transplanted donor tumors. Another approach to render the WAP-Myc tumor cells more metastatic is to introduce additional mutations or changes in the expression of candidate genes. For example, a previous study showed that overexpression of the pro-angiogenic factor VEGF in the MMTV-MYC mammary tumor model resulted in much higher pulmonary metastasis rates^42^.

We have previously used the WAP-Myc syngraft model to demonstrate a new therapeutic strategy for MYC-high breast cancer^25^. A combination of the Bcl-2 inhibitor venetoclax and anti-diabetic drug metformin induced cell death specifically in MYC-high tumors, and this response was augmented by checkpoint inhibitor anti-PD-1, which prevents dampening the T-cell-mediated immune response through PD-1/PD-L1 (Programmed death-ligand 1) interaction. In our current study, we found increased infiltration of T cell populations in the metastatic tumors compared to the primary tumors (Fig. 4a,b), while other immune cell types showed similar levels of infiltration. In addition, cytotoxic T cells infiltrating in WAP-Myc metastases express higher levels of interferon gamma compared to primary cells (Fig. 4c). Interferon gamma can play multiple roles in cancer, both pro- and anti-tumorigenic, from increasing cytotoxicity and motility^43^ to downregulation of major histocompatibility complexes and upregulation of checkpoint inhibitors such as PD-L1^44^. These data could suggest that MYC-driven lung metastasis might benefit from treatments that reinvigorate the immune system, for example with a drug combo that combines induction of cancer cell killing and immune checkpoint blockade such as the aforementioned combination of venetoclax, metformin and anti-PD-1^25^. Further studies will be needed to test this hypothesis.

Classically, T cells have been thought as tumor suppressive, however, certain subtypes of T helper cells, such as regulatory T cells or pro-inflammatory IL-17-expressing Th17 cells, have immune suppressive functions in tumors and promote metastasis to the lungs^45^. As the immunoprofiling panels used in this study do not cover all immune cell types, it is important to characterize the T cell infiltrates and cytokine profiles in metastatic tumors and determine their function in prospective studies.

In summary, we have developed a robust WAP-Myc mammary tumor transplant mouse model that shows moderate spontaneous autochthonous metastasis to lungs. This model can be used to study the mechanisms of metastasis in MYC-driven breast cancer in future studies.

## Data Availability

The datasets generated during and/or analysed during the current study are available from the corresponding author on reasonable request.
